# Prognostic value of a new computed tomography severity score in hemorrhagic fever with renal syndrome

**DOI:** 10.1007/s10140-025-02322-9

**Published:** 2025-03-12

**Authors:** Se Woo Kim, Cheong-Il Shin, Min Woo Kang, Min Cheol Kim, Donghwan Kim

**Affiliations:** 1 Department of Radiology, Armed Forces Capital Hospital, Gyeonggi-do, Republic of Korea; 2 Department of Radiology, Seoul National University Hospital, 101, Daehak-ro, Jongno-gu, Seoul, 03080 Republic of Korea; 3 Department of Radiology, Seoul National University College of Medicine, 101, Daehak-ro, Jongno-gu, Seoul, 03080 Republic of Korea; 4 Department of Internal Medicine, Armed Forces Daejeon Hospital, Daejeon, Republic of Korea; 5 Department of Internal Medicine, Armed Forces Capital Hospital, Gyeonggi-do, Republic of Korea

**Keywords:** Hemorrhagic fever with renal syndrome, Hantavirus, Computed tomography, Proteinuria, Renal replacement therapy

## Abstract

**Purpose:**

To develop of a novel computed tomography (CT) severity score for hemorrhagic fever with renal syndrome (HFRS) and evaluate its correlation with disease severity and adverse outcomes.

**Methods:**

This retrospective study included 37 patients diagnosed with HFRS from January 2012 to December 2023 who had available clinical laboratory and abdominal CT data during the acute phase. The CT severity score (range 0–5) was based on perirenal fat stranding, pararenal fascia thickening, anterior pararenal space fat stranding, ascites, and pleural effusion. Correlations between the score and markers of inflammation, thrombocytopenia, proteinuria, and adverse outcomes—including nephrotic range proteinuria and renal replacement therapy (RRT)—were analyzed.

**Results:**

The CT severity score exhibited moderate to strong correlations with markers of inflammation (white blood cell count, ρ = 0.65, *p* < 0.001), thrombocytopenia (platelet count, ρ = -0.54, *p* < 0.001), and proteinuria (urine protein-to-creatinine ratio, ρ = 0.56, *p* < 0.001). Higher scores were associated with increased nephrotic range proteinuria in Chi-squared test for trend (*p*-for-trend = 0.001). A one-point increase in the score significantly increased odds of requiring RRT in logistic regression analysis (odds ratio: 9.89, *p* = 0.047). The score achieved an area under the receiver operating characteristics curve of 0.819 for predicting RRT.

**Conclusion:**

The CT severity score correlates well with disease severity and adverse outcomes in HFRS and can be assessed using noncontrast CT, making it a valuable prognostic tool in young male population. Further validation in diverse populations is warranted.

**Supplementary Information:**

The online version contains supplementary material available at 10.1007/s10140-025-02322-9.

## Introduction

Hemorrhagic fever with renal syndrome (HFRS) is a hantavirus-caused clinical syndrome characterized by a febrile illness accompanied by acute kidney injury (AKI) and thrombocytopenia [1]. Unlike hantavirus cardiopulmonary syndrome, another hantavirus disease primarily occurring in the New World, HFRS affects approximately 200,000 people annually in the Old World, including Russia, Northern Europe, China, and Korea [2, 3]. The infection is more common among young adults aged 20 to 40 years, with a male predilection; reported male-to-female ratios range from 2:1 to 3:1 worldwide [3]. Occupational exposure to infected rodents contributes to higher risk among military recruits, forestry workers, and farmers [1–3]. The mortality rate of this disease varies from less than 1% to 10–15%, depending on the type of causative virus species [1].

Given the high mortality rate associated with HFRS, numerous studies have investigated prognostic indicators, with most focusing on laboratory test results [4–9]. A few studies have highlighted certain sonographic findings, such as an elevated renal resistive index, fluid third-spacing, and gallbladder wall thickening, as being associated with disease severity [10, 11]. In the context of computed tomography (CT), nonspecific findings such as perirenal fat stranding, pararenal fascia thickening, and fluid third-spacing have been recognized as features of HFRS [12, 13]. However, to the best of our knowledge, no studies have linked CT findings to disease prognosis, limiting the role of radiologists in this area.

In this study, we developed a new HFRS severity scoring system based on abdominal CT findings and evaluated the possible correlation with patient outcomes.

## Materials and methods

The institutional review board of Armed Forces Medical Command approved this retrospective observational study (IRB Number: AFMC 2024-01-007). The requirement for informed patient consent was waived due to the retrospective nature of the study.

### Study population

**Fig. 1:**
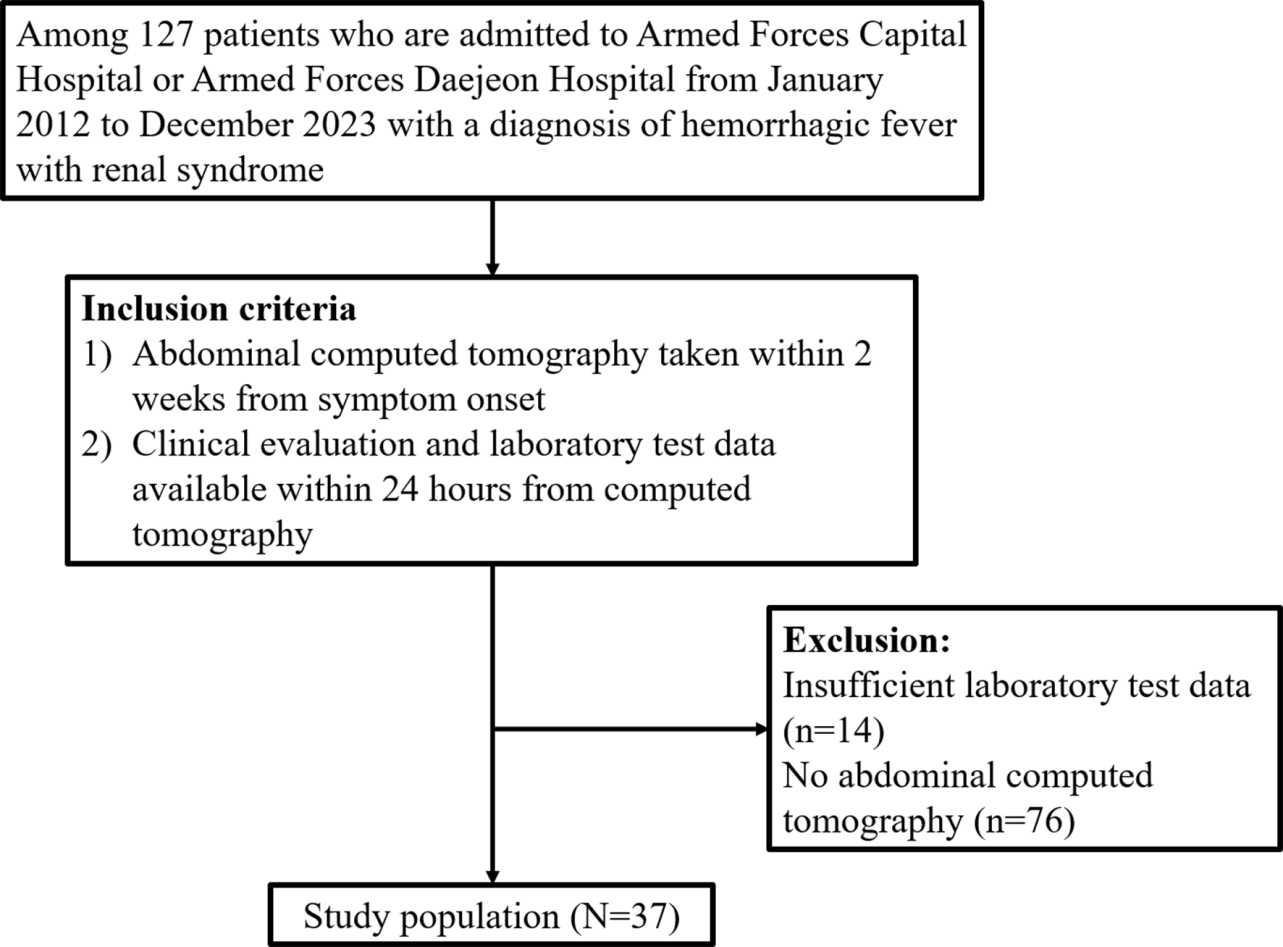
Flow diagram of study population enrollment

Among 127 patients admitted to Armed Forces Capital Hospital and Armed Forces Daejeon Hospital with a diagnosis of HFRS from January 2012 to December 2023, those meeting the following inclusion criteria were consecutively enrolled: (1) Abdominal CT taken within 2 weeks of the symptom onset, and (2) Availability of the following data within 24 h from the CT examination: review of systems, physical examination, complete blood count, blood urea nitrogen, serum creatinine, serum total protein, serum albumin, serum total bilirubin, serum aspartate/alanine aminotransferase, serum alkaline phosphatase, serum electrolytes, serum C-reactive protein (CRP), and urine protein-to-creatinine ratio (uPCR). After excluding 76 patients without abdominal CT and 14 patients with missing laboratory test data, 37 patients were finally enrolled. All study participants were active-duty members of the Republic of Korea Armed Forces, and baseline renal function was confirmed to be a Modification of Diet in Renal Disease (MDRD)-estimated glomerular filtration rate (eGFR) of 60 ml/min/1.73m^2^ or higher, based on pre-enlistment examinations. However, the exact results of the pre-enlistment examinations were not disclosed and therefore could not be determined. Figure 1 presents the flowchart of the patient inclusion process.

### Collection of clinical data and laboratory test results

The electronic medical records of the study population were thoroughly reviewed by an author (S.W.K). Baseline demographics and the clinical and laboratory data closest to the time of CT were recorded. Clinical signs and symptoms were classified under the following clusters of manifestations: Flu-like (fever, headache, myalgia, cough, rhinorrhea, sore throat), gastrointestinal (anorexia, nausea, vomiting, diarrhea, constipation), hemorrhagic (petechiae, hemorrhage of head and neck region or other body parts), renal (altered urine output, edema), abdominal pain (subjective pain, clinician-determined tenderness), and back pain (subjective pain, clinician-determined costovertebral angle tenderness).

### Image interpretation

All image interpretations were done by two board-certified radiologists (M.C.K and S.W.K, with 5 and 8 years of experience in abdominal radiology, respectively) in consensus. If a discrepancy occurred, another expert radiologist (C-I.S, with 20 years of experience in abdominal imaging) reviewed the images. On noncontrast images, the presence or absence of juxta-renal findings—perirenal fat stranding, pararenal fascia thickening, and anterior pararenal space fat stranding—, fluid third-spacing—ascites and pleural effusion—, and ancillary features, such as pulmonary interstitial thickening and pancreatic swelling, were assessed [12, 13]. The pole-to-pole lengths of both kidneys were also measured [12, 13].

Perirenal and anterior pararenal space fat stranding were defined as any fat stranding that did not correspond to normal vasculature. For pararenal fascia thickening, a cutoff of 2 mm was applied, as this structure is often visible as a thin linear band even in normal patients. For patients who underwent portal venous phase (PVP) imaging, the presence or absence of periportal hypodensity and gallbladder wall thickening was also evaluated [13]. A schematic diagram of the retroperitoneal space, along with representative images of perirenal fat stranding, pararenal fascia thickening, and anterior pararenal space fat stranding, is depicted in Electronic supplementary material 1, 2 and 3.

### HFRS CT severity score

Based on the CT findings, a novel scoring system for HFRS disease severity (range 0–5) was developed as follows (Table 1):

**Table 1:** CT severity score calculation

	Findings	Score
**If** perirenal fat stranding^†^ is present	Perirenal fat stranding^†^	+ 1
	Pararenal fascia thickening^‡^	+ 1
	Anterior pararenal space fat stranding^†^	+ 1
	Ascites	+ 1
	Pleural effusion	+ 1
**If** perirenal fat stranding^†^ is absent	Any findings	0

†Any fat stranding that does not correspond to normal vasculature

‡Pararenal fascia thickness > 2 mm

The reason for not assigning a score when perirenal fat stranding is absent is to enhance specificity by excluding fluid third-spacing that may arise from non-renal causes

1. If perirenal fat stranding is absent, CT severity score is 0.
2. If perirenal fat stranding is present, add 1 point for presence of each of the following findings: perirenal fat stranding, pararenal fascia thickening, anterior pararenal space fat stranding, ascites, and pleural effusion.

The reason for not assigning a score when perirenal fat stranding is absent is to enhance specificity by excluding fluid third-spacing that may arise from non-renal causes.

### Definition of adverse clinical outcomes

Any death and severe AKI requiring renal replacement therapy (RRT) were recorded as adverse outcome [1, 5, 14, 15]. Additionally, the presence of nephrotic range proteinuria, which is defined as uPCR > 3.0 g/g, was also recorded as an indicator of disease severity and a risk factor for adverse outcome [14–17].

### Statistical analysis

To establish biological plausibility and relevance, the correlation between the CT severity score and laboratory test results was assessed using Spearman’s correlation analysis. The prevalence of nephrotic range proteinuria according to the CT severity scores was evaluated, and the Chi-square test for trend was performed. Univariable logistic regression analysis was conducted to evaluate the potential association between CT severity score and adverse outcomes. Other known risk factors for severe AKI or poor prognosis — eGFR, proteinuria, thrombocytopenia, hypoalbuminemia, hyperbilirubinemia, hyponatremia, and elevated CRP — were also evaluated [5–9, 17]. The receiver operating characteristics (ROC) curve analysis was done to evaluate the value of the CT severity score as an indicator of adverse outcomes.

A value of *p* < 0.05 was considered to denote a statistically significant difference. All statistical analysis was performed using commercially available statistical software (MedCalc for Windows, version 22.017, MedCalc Software).

## Results

### Demographics, clinical manifestations, and laboratory test results

All patients were male, and most were in their early twenties (mean ± standard deviations [years]: 20.7 ± 1.4). Nine patients diagnosed through polymerase chain reaction were confirmed to be infected with Hantaan virus. Because the remaining patients were diagnosed by immunofluorescence assay, the specific causative pathogen could not be identified. For all patients in study population, there were no documented underlying conditions, such as diabetes mellitus, hypertension, or urinary tract infection, nor any other comorbidities that could have significantly affected the patient’s clinical course, particularly renal function. All patients presented with fever. The proportion of patients presenting with gastrointestinal manifestations was 81.1% (30/37). Abdominal pain and back pain were often present in the patients (abdominal pain: 59.5% [22/37]; back pain: 48.6% [18/37]). Renal manifestations occurred in 37.8% (14/37) of the patients. For complete blood count, 97.3% (36/37) of patients showed thrombocytopenia, and 64.9% (24/37) of patients showed leukocytosis. Blood urea nitrogen and serum creatinine were high, and eGFR was low. Table 2 summarizes the results. Electronic supplementary material 4 summarizes detailed frequencies of specific symptoms and signs within each cluster of manifestations.

**Table 2:** Demographics, clinical manifestations, and laboratory results of the study population

**Demographics and basic characteristics**	
Age (years)	20.7 ± 1.4
Sex (M: F)	37:0
**Cluster of manifestations**	
Flu-like manifestations	100.0 (37/37)
Gastrointestinal manifestations	81.1 (30/37)
Hemorrhagic manifestations	27.0 (10/37)
Renal manifestations	37.8 (14/37)
Abdominal pain	59.5 (22/37)
Back pain	48.6 (18/37)
**Laboratory tests**	
Hemoglobin (g/dL) ^†^	15.6 ± 2.1
White blood cell (x1000/uL) ^†^	12.9 ± 6.8
-Leukocytosis	64.9 (24/37)
-Leukocytopenia	5.4 (2/37)
Platelet (x1000/uL) ^†^	62.5 ± 50.5
-Thrombocytopenia	97.3 (36/37)
Blood urea nitrogen (mg/dL) ^†^	30.8 ± 24.6
Serum creatinine (mg/dL) ^†^	2.6 ± 2.4
eGFR (mL/min/1.73m^2^) ^†^	64.8 ± 40.7
Serum total protein (g/dL) ^†^	5.8 ± 0.6
Serum albumin (g/dL) ^†^	3.3 ± 0.5
Total bilirubin (mg/dL) ^†^	0.6 ± 0.3
Alkaline phosphatase (IU/L) ^†^	170.3 ± 113.3
Aspartate aminotransferase (IU/L) ^†^	110.1 ± 57.4
Alanine aminotransferase (IU/L) ^†^	82.7 ± 56.0
C-reactive protein (mg/dL)	5.1 ± 2.7
Sodium (mg/dL)	136.8 ± 5.1
Potassium (mg/dL)	4.0 ± 0.5
Urine protein-to-creatinine ratio (g/g) ^†^	4.66 ± 4.17

Data are percentages. Data in parenthesis are numbers used to calculate percentages

†Data are arithmetic means ± standard deviations

**eGFR**, estimated glomerular filtration rate;

### Abdominal CT findings in study population

**Fig. 2:**
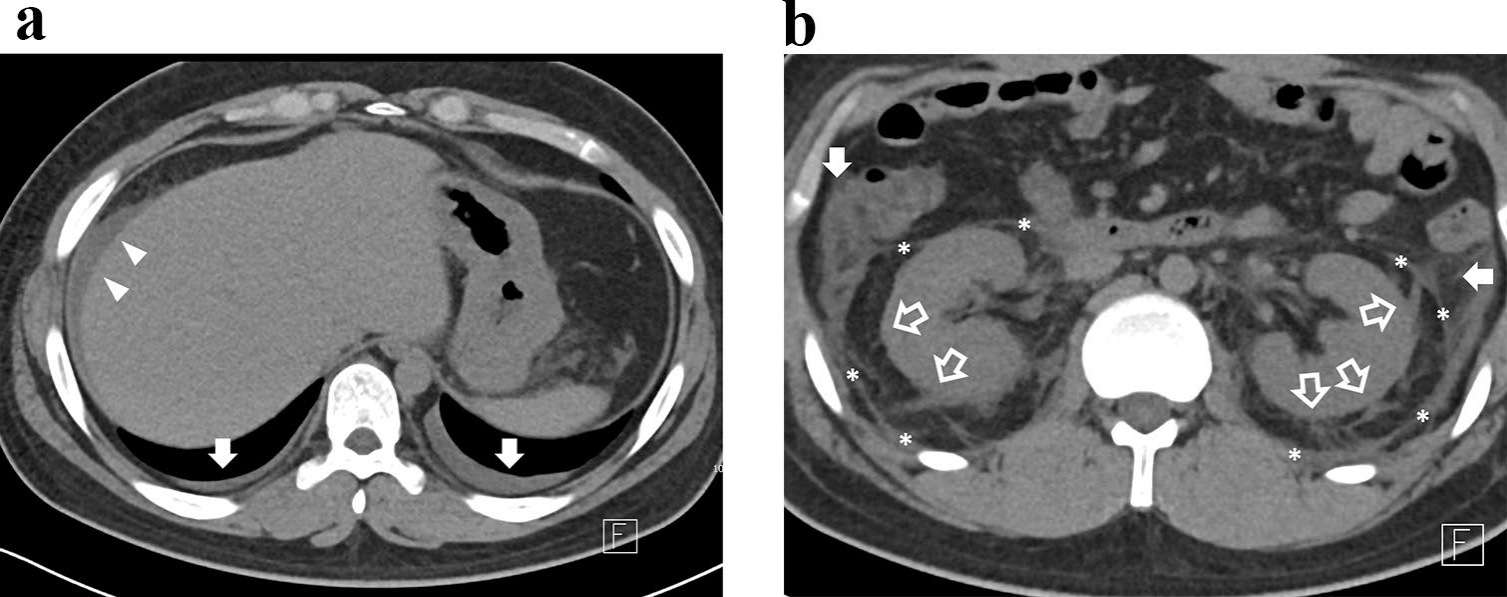
Representative CT image of a 20-year-old male patient with hemorrhagic fever with renal syndrome. **A**. At the diaphragm level, ascites (white arrowheads) and bilateral pleural effusion (white arrows) are noted. **B**. At the kidney level, perirenal fat strandings (empty arrows), pararenal fascia thickenings (asterisks) and anterior pararenal fat strandings (white arrows) are noted. The CT severity score was 5. Patient had nephrotic range proteinuria (urine protein-to-creatinine ratio of 18.7 g/g) at the time of CT examination. Hemodialysis was initiated three days following the CT scan

**Fig. 3:**
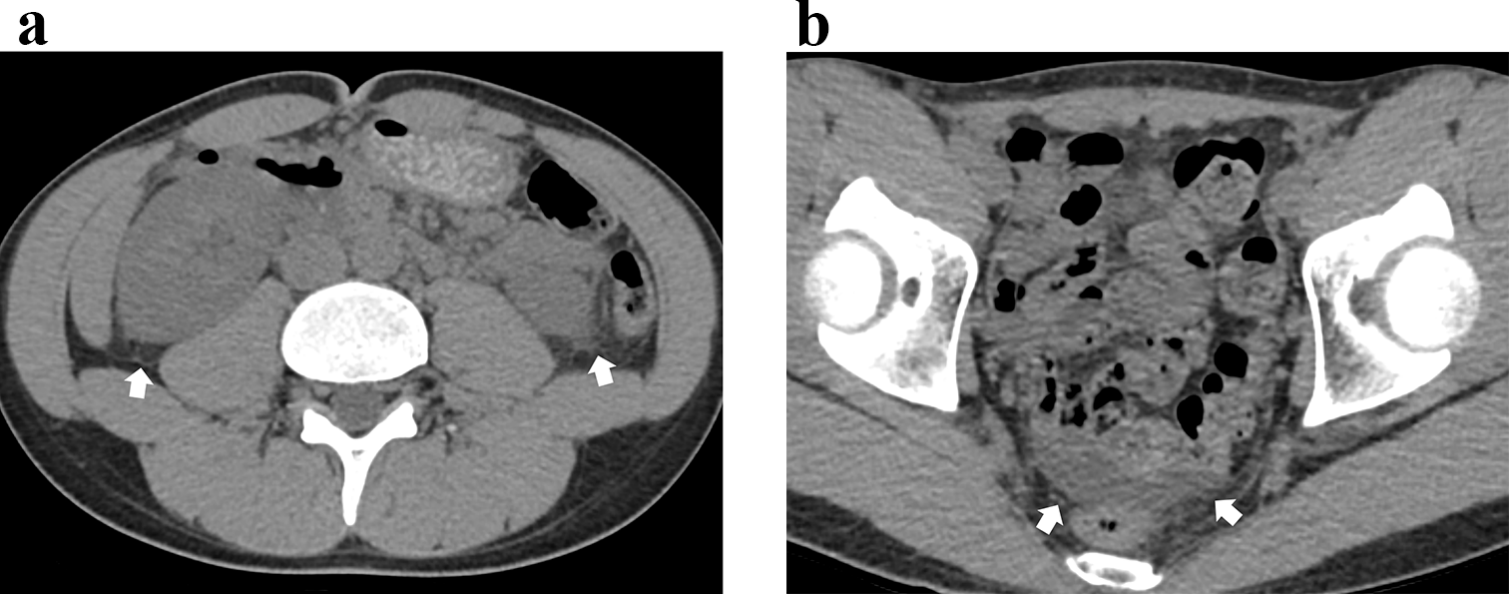
Representative CT image of a 20-year-old male patient with hemorrhagic fever with renal syndrome. **A**. At the kidney level, subtle perirenal fat strandings are noted (white arrows). **B**. At the level of pelvis, small amount of pelvic ascites is present (white arrows). The CT severity score was 2. Patient had no nephrotic syndrome (urine protein-to-creatinine ratio of 0.4 g/g) at the time of CT examination, and did not require renal replacement therapy throughout the hospital course

The median (interquartile range) time [days] from symptom onset to CT was 5 (3–7). Of the 37 patients, 23 patients underwent IV contrast-enhanced CT, and 14 patients underwent non-contrast CT only. Frequent CT findings near the kidneys were classified as juxta-renal findings—perirenal fat stranding (91.9% [34/37]), pararenal fascia thickening (86.5% [32/37]), and anterior pararenal space fat stranding (67.6% [25/37]). Third-space fluid losses such as ascites (94.6% [35/37]) and pleural effusion (40.5% [15/37]) were also frequently demonstrated. Table 3 summarizes the results of CT interpretation, including the prevalence of other findings and results of renal pole-to-pole length measurements that are not mentioned in the text. No patient presented with hydronephrosis. Figures 2 and 3 show representative case images of patients with high and low CT severity scores.

**Table 3:** Abdominal computed tomography findings of study population

**Juxta-renal findings**	
Perirenal fat stranding	91.9 (34/37)
Pararenal fascia thickening	86.5 (32/37)
Anterior pararenal space fat stranding	67.6 (25/37)
**Third-spacing of fluid**	
Ascites	94.6 (35/37)
Pleural effusion	40.5 (15/37)
**CT severity score**	3.8 ± 1.4
**Other findings**	
Pancreas swelling	8.1 (3/37)
Pulmonary interstitial thickening	8.1 (3/37)
Periportal hypodensity on PVP	47.8 (11/23)
Gallbladder wall thickening on PVP	21.7 (5/23)
**Renal pole to pole length (mm)**	
Right kidney^†^	114.8 ± 7.9
Left kidney^†^	118.3 ± 7.7

Data are percentages and data in parentheses are numbers used to calculate percentages

†Data are arithmetic means ± standard deviations

**CT**, computed tomography; **PVP**, portal venous phase;

### Correlation between CT severity score and laboratory tests

The CT severity score demonstrated a strong positive correlation with white blood cell count (Spearman’s ρ = 0.65, *p* < 0.001). It also showed moderate positive correlation with uPCR (Spearman’s ρ = 0.56, *p* < 0.001) and moderate negative correlations with platelet count (Spearman’s ρ = -0.54, *p* < 0.001), serum total protein (Spearman’s ρ = -0.47, *p* = 0.004), and serum albumin (Spearman’s ρ = -0.56, *p* < 0.001). There were no significant correlations with other variables, such as eGFR, sodium, or CRP. Table 4 summarizes the results of correlation analyses.

**Table 4:** Correlation between CT severity score and laboratory tests

	Spearman’s ρ [95% confidence interval]	*p*
Hemoglobin (g/dL)	0.20 [-0.14, 0.49]	0.241
White blood cell (x1000/uL)	0.65 [0.41, 0.80]	***< 0.001***
Platelet (x1000/uL)	-0.54 [-0.74, -0.27]	***< 0.001***
Blood urea nitrogen (mg/dL)	0.08 [-0.26, 0.39]	0.659
Serum creatinine (mg/dL)	0.16 [-0.17, 0.46]	0.338
eGFR (mL/min/1.73m^2^)	-0.16 [-0.46, 0.17]	0.348
Serum total protein (g/dL)	-0.47 [-0.69, -0.17]	***0.004***
Serum albumin (g/dL)	-0.56 [-0.75, -0.29]	***< 0.001***
Total bilirubin (mg/dL)	0.04 [-0.29, 0.36]	0.836
Alkaline phosphatase (IU/L)	-0.01 [-0.33, 0.31]	0.950
Aspartate aminotransferase (IU/L)	0.23 [-0.11, 0.51]	0.179
Alanine aminotransferase (IU/L)	0.26 [-0.07, 0.54]	0.123
Serum sodium (mg/dL)	-0.12 [-0.43, 0.21]	0.467
Serum potassium (mg/dL)	-0.03 [-0.35, 0.29]	0.846
Serum C-reactive protein (mg/dL)	0.11 [-0.23, 0.42]	0.531
Urine protein-to-creatinine ratio (g/g)	0.56 [0.29, 0.75]	***< 0.001***

***Bold italics*** indicate statistical significance

**eGFR**, estimated glomerular filtration rate;

### Prevalence of adverse clinical outcomes

There were no deaths in the study population. About two-thirds (62.2% [23/37]) of the patients had nephrotic range proteinuria at the time of CT examination, and 13.5% (5/37) received RRT (four patients received intermittent hemodialysis, and one patient received continuous RRT).

### Association between CT severity score and nephrotic range proteinuria

**Fig. 4:**
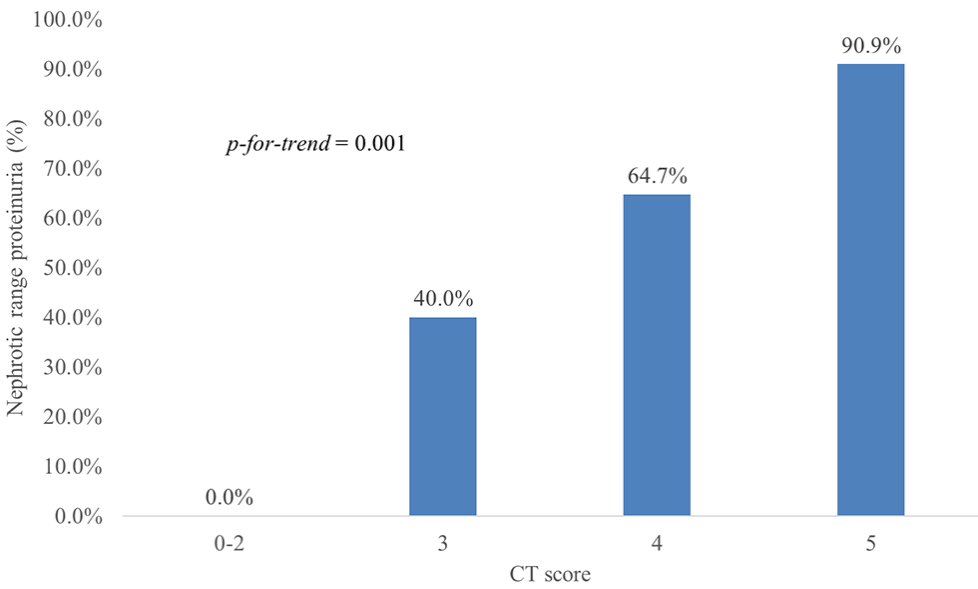
Prevalence of nephrotic range proteinuria according to the CT severity score

The proportion of patients with nephrotic range proteinuria was 0.0% (0/4), 40.0% (2/5), 64.7% (11/17), and 90.9% (10/11) for subgroups with CT severity scores of 0–2, 3, 4, and 5, respectively. The prevalence of nephrotic range proteinuria tended to increase with higher CT severity scores (*p*-for-trend = 0.001). Figure 4 shows the prevalence of nephrotic range proteinuria according to the CT severity score.

### Association of CT severity score and requirement for renal replacement therapy

Univariable logistic regression analyses were conducted to evaluate the association between the clinical outcomes and the CT severity score, along with other known risk factors for poor prognosis. Among the variables analyzed, the CT severity score was the only statistically significant factor. A one-point increase in CT severity score was associated with significantly higher odds of receiving RRT (odds ratio [95% confidence interval]: 9.89 [1.03–94.87], *p* = 0.047).

**Fig. 5:**
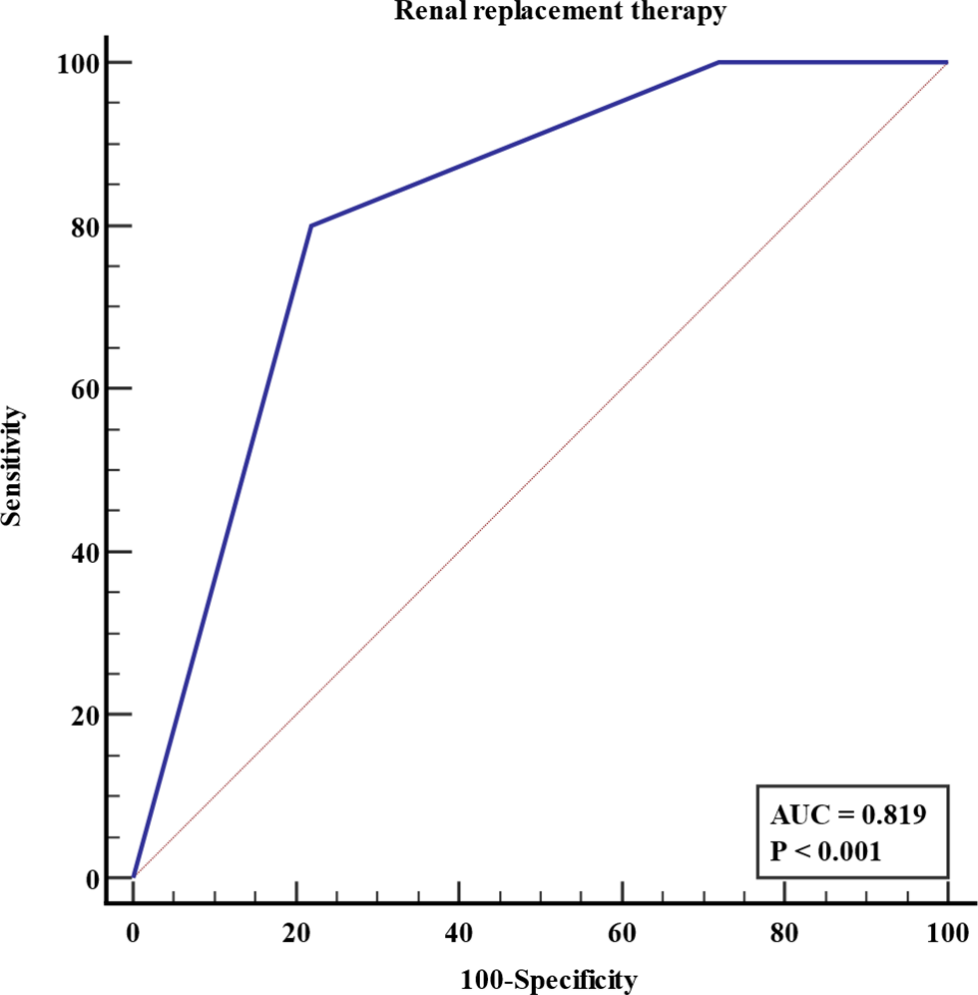
Receiver operating characteristics curve of CT severity score for prediction of severe acute kidney injury requiring renal replacement therapy (area under curve = 0.819). The CT severity score of 5 could predict need for renal replacement therapy with 80.0% sensitivity and 78.1% specificity in the study population

Due to the absence of other variables with statistical significance, multivariable analysis was not conducted. Table 5 shows the results of the logistic regression analyses. In the ROC curve analysis, the area under the ROC curve for the CT severity score in predicting RRT was 0.819 (95% confidence interval: 0.658–0.926). Figure 5 illustrates the ROC curves.

**Table 5:** Logistic regression analyses of risk factors for renal replacement therapy

	Odds ratio [95% confidence interval]	*p*
CT severity score	9.89 [1.03, 94.87]	***0.047***
eGFR (mL/min/1.73m2)	0.98 [0.95, 1.01]	0.136
Urine protein-to-creatinine ratio (g/g)	1.12 [0.92, 1.36]	0.247
Platelet (x1000/uL)	0.95 [0.91, 1.00]	0.064
Serum albumin (g/dL)	0.15 [0.01, 2.28]	0.174
Total bilirubin (mg/dL)	0.37 [0.01, 14.62]	0.598
Serum sodium (mg/dL)	0.92 [0.76, 1.11]	0.384
Serum C-reactive protein (mg/dL)	1.07 [0.76, 1.50]	0.709

***Bold italics*** indicate statistical significance

Odds ratio represents the change in the likelihood of renal replacement therapy per one-unit increase

**eGFR**, estimated glomerular filtration rate;

## Discussion

The results of our study align with previously published reports on the clinical features and imaging findings of HFRS [1, 2, 10–13]. Febrile manifestations, abdominal pain, back pain, and oliguric AKI were frequently observed in our study population, consistent with earlier studies [1]. Regarding imaging findings, juxta-renal involvement and fluid third-spacing were prominent features [12, 13]. While renal swelling is a recognized imaging finding in HFRS, no standardized criterion exists for determining nephromegaly based on pole-to-pole kidney length. In our study, the mean pole-to-pole lengths of the right and left kidneys were 114.8 mm and 118.3 mm, respectively, which are smaller than the corresponding measurements of 125.7 mm and 127.8 mm reported in a Belgian hospital [13]. Variations in body habitus and ethnicity between the study populations may have contributed to this discrepancy.

The CT severity score was developed based on careful observation of common imaging features previously described in the literature [10–13]. Notably, the features classified as juxta-renal findings appeared in a stepwise progression: all patients with anterior pararenal fat stranding also demonstrated pararenal fascia thickening, and all with pararenal fascia thickening exhibited perirenal fat stranding. This unique imaging spectrum underscores the progressive nature of juxta-renal involvement, ranging from isolated perirenal fat stranding to the combined presence of all three findings.

Although the CT severity score was developed solely based on imaging findings, it demonstrated moderate to strong correlations with laboratory markers of inflammation (white blood cell count), thrombocytopenia (platelet count), and proteinuria (uPCR, albumin, and total protein). This indicates that the CT severity score effectively reflects the underlying disease process. Furthermore, previous studies have identified hypoalbuminemia, proteinuria, and thrombocytopenia as markers of disease severity, providing additional validation for the CT severity score as a reliable indicator of the clinical manifestations of HFRS [5, 6, 8, 9].

However, the components of the CT severity score are highly nonspecific imaging features, warranting careful interpretation. Fluid third-spacing can result from a variety of causes, including malignancy, inflammation, renal insufficiency, heart failure, and portal hypertension [18]. To enhance the specificity of the CT severity score, fluid third-spacing was excluded when juxta-renal findings were absent. Nevertheless, juxta-renal findings themselves can also arise from diverse etiologies, such as renal inflammation, infection, systemic diseases, fluid overload, or bladder outlet obstruction [19, 20]. Notably, the observation that juxta-renal findings can result from fluid overload raises the possibility that the CT score’s prognostic value may, in part, reflect the detection of aggressive resuscitation efforts in patients already experiencing clinical deterioration. Severe fluid overload is also a major indication for renal replacement therapy (RRT) [21]. Ideally, the confounding effect of fluid overload should have been accounted for; however, due to the retrospective nature of this study, precise input and output monitoring before and during hospitalization was unavailable for most patients. This limitation represents a significant drawback of our study. Additionally, our study population consisted exclusively of young male military personnel in their twenties, which may have led to an overestimation of the utility of the CT severity score. For broader generalizability, further validation studies are needed, incorporating patients of varying ages and genders.

In our study population, the CT severity score demonstrated a significant correlation with nephrotic range proteinuria. Hantavirus species that cause HFRS have a tropism for renal capillaries [1, 2, 22]. After entering the endothelial cells of renal capillaries, the virus activates various pathways to release vasoactive factors, resulting in increased vascular permeability, platelet activation, and an overreacting host immune response. In addition to endothelial cells, podocytes and tubular epithelial cells can also be infected by the virus, resulting in massive proteinuria [22]. The degree of proteinuria is known to be related to the severity of the disease, but excess protein in the urine itself can also cause nephrotoxicity and exacerbate kidney damage [5, 14, 17, 22]. Thus, it is noteworthy that the CT severity score correlates well with the degree of proteinuria, reflecting its potential as a reliable indicator of disease severity.

There are several reasons why RRT was selected as a clinical outcome. First, RRT is generally indicated for severe AKI, so the use of RRT itself was considered to indicate serious renal damage [14, 21, 23]. Second, there are previous reports claiming that the optimal timing of RRT improves prognosis in patients with severe AKI compared with delayed RRT application [23]. Therefore, using the CT severity score to predict future RRT requirements in advance has the potential to help physicians prepare for RRT at the appropriate time, thereby improving patient prognosis. Third, regardless of the timing of RRT, access to this advanced treatment is often unavailable in resource-limited hospitals. Early risk stratification using the CT severity score could help identify patients likely to require RRT, allowing for timely transfer to referral hospitals before clinical deterioration occurs, ultimately benefiting patient prognosis.

In previous studies on prognostic factors in HFRS that included more than 100 patients, several laboratory variables—C-reactive protein, total bilirubin, albumin, thrombocytopenia, and proteinuria—were reported as significant prognostic markers [6, 7, 9]. However, in our study, these variables did not demonstrate statistically significant associations with HFRS prognosis in univariable analysis. Given the relatively small sample size of our study (*n* = 37), it is plausible that repeating the analysis in a larger population could yield results more consistent with previous literature, where a greater number of clinico-laboratory variables reached statistical significance in univariable analysis. A larger study would also allow for a multivariable model with multiparametric adjustment, which could help determine whether the CT severity score remains an independent prognostic indicator even after controlling for other significant factors. Therefore, further research with a larger population is necessary to validate these findings.

Abdominal CT is not routinely performed in patients with suspected HFRS, likely due to insufficient evidence supporting its diagnostic or prognostic value to justify the radiation exposure and, especially in the case of contrast-enhanced studies, the potential nephrotoxicity of iodinated contrast agents [14]. This study, however, indicates that the CT findings identified as prognostically significant can be evaluated without the use of iodinated contrast agents. In our study population, more than half of the patients underwent contrast-enhanced CT. Due to the retrospective nature of this study, it was not possible to determine a clear justification for the use of contrast agents. Even if contrast administration was unnecessary, our findings demonstrate the potential utility of noncontrast CT in HFRS management. With modern dose-reduction strategies, low-dose noncontrast abdominal CT could be a reasonable option for evaluating patients with or suspected of having HFRS [24, 25].

This study has several limitations that should be considered. First, the retrospective design may have introduced selection biases. The absence of randomization and standardized imaging protocols could have affected the generalizability of the results. Second, the study population consisted exclusively of young male military personnel, which may limit the applicability of the findings to broader populations, including females, older adults, and individuals with diverse comorbidities. The fact that overall patient outcomes were very good compared to the known high mortality rate of HFRS would probably be attributable to the extremely biased study population [1, 2]. Third, only 24.3% (9 out of 37) of the study population had a definitive identification of the causative pathogen through polymerase chain reaction, confirming Hantaan virus disease. Immunofluorescence assay, used for the remaining patients, cannot differentiate between viruses within the orthohantavirus genus. South Korea is an endemic area for both Seoul virus and Hantaan virus [2]. Potential differences in imaging findings and outcomes between different viruses were not addressed in the current study [1, 2]. Fourth, while the CT severity score demonstrated significant correlations with markers of inflammation, thrombocytopenia, and proteinuria, its nonspecific imaging features require cautious interpretation, as these findings can also arise from non-HFRS conditions [18–20]. Finally, the small sample size may have limited the statistical power. Future studies involving larger, more diverse populations and prospective designs are needed to validate and refine the CT severity score for broader clinical application.

In conclusion, this study introduces a novel CT severity score for HFRS that correlates with degree of proteinuria and the need for renal replacement therapy. The score’s reliance on noncontrast imaging makes it a safer option for risk stratification, particularly in resource-limited settings. Further validation in larger, and more diverse populations is needed to confirm its clinical utility.

## Electronic supplementary material

Below is the link to the electronic supplementary material.

Supplementary Material 1

## Data Availability

The data that support the findings of this study are not openly available due to reasons of sensitivity, and are available from the corresponding author upon reasonable request.
