# Supplementary material for: Prognostic value of a new computed tomography severity score in hemorrhagic fever with renal syndrome
Source: Emerg Radiol. 2025 Mar 12;32(3):377–85. doi: 10.1007/s10140-025-02322-9 (PMC12133915; doi:10.1007/s10140-025-02322-9)
Supplement: Supplementary file 1 — Supplementary Material 1 [file 10140_2025_2322_MOESM1_ESM.pdf]

Electronic Supplementary Material

Online Resource 1

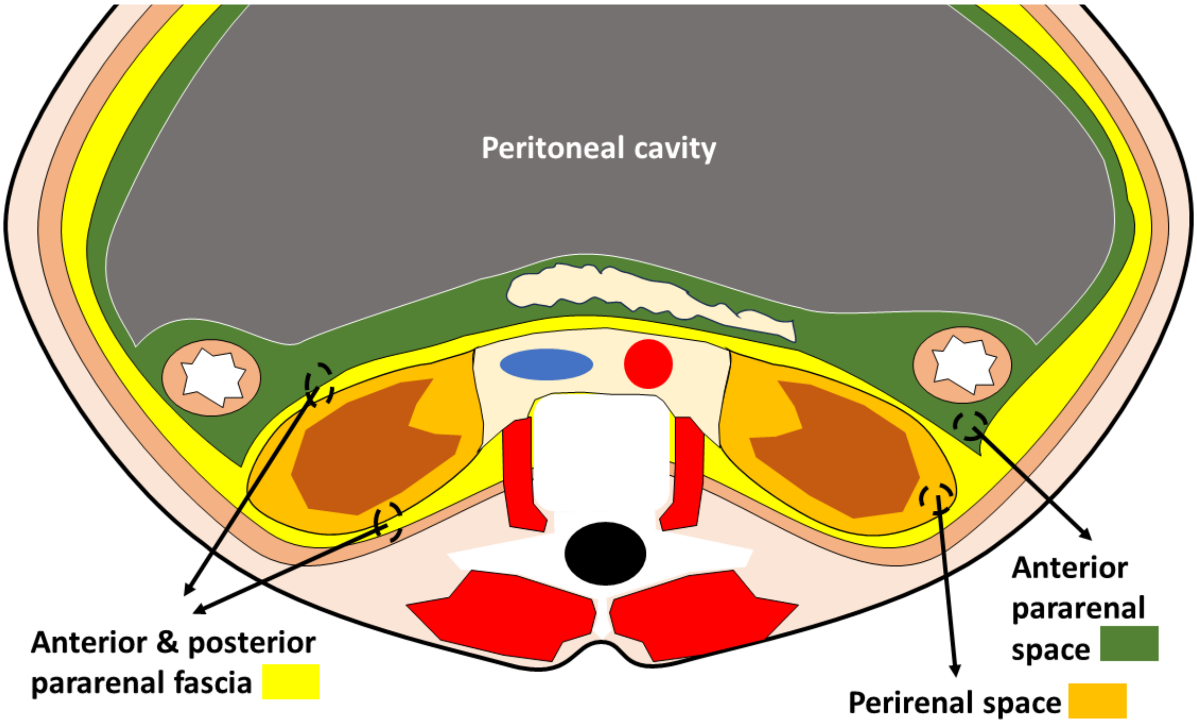

Online Resource 2

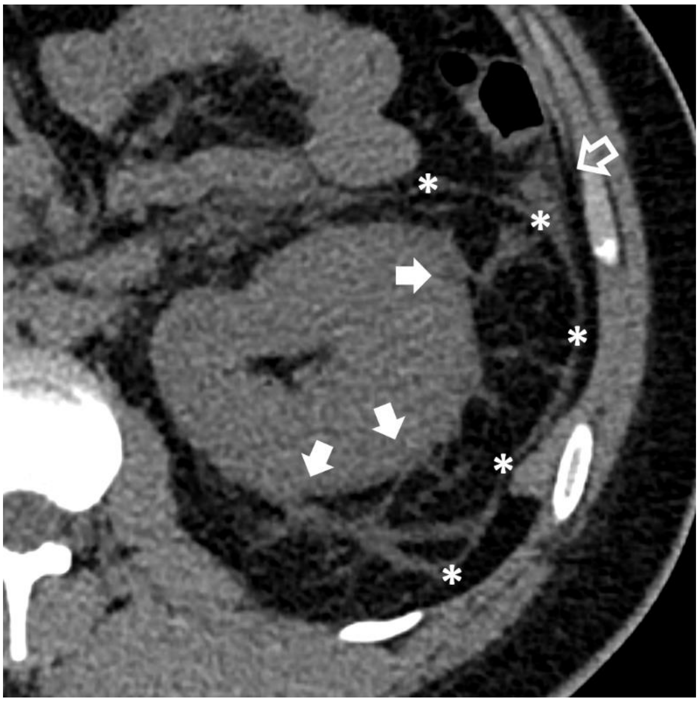

Online Resource 3

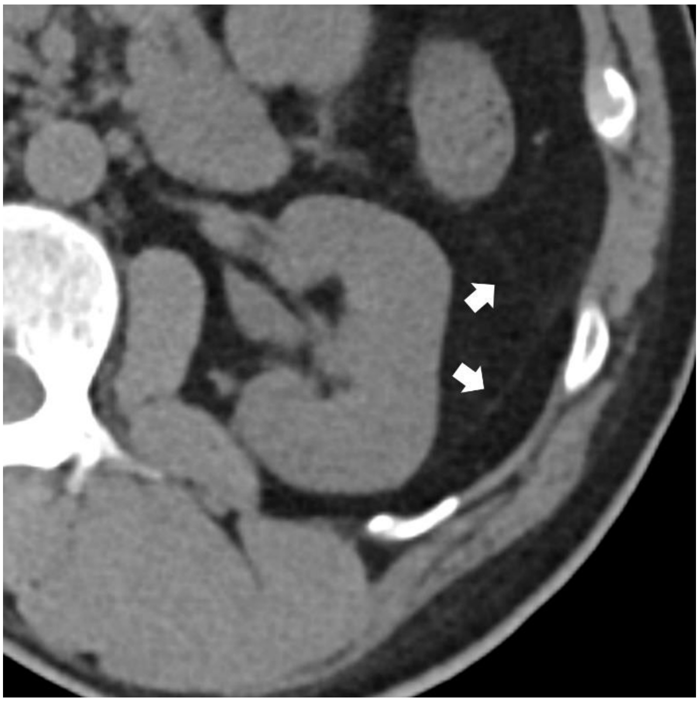

Online Resource. **1**. Schematic drawing describing anatomy of retroperitoneal spaces and fascia. **2**. Representative image showing the perirenal fat strandings (white arrows), pararenal fascia thickenings (asterisks), and anterior pararenal space fat stranding (empty arrow). **3**. Representative image showing no juxta-renal findings. Note that pararenal fascia less than 2mm is normally seen as thin linear structure (white arrows)

Online Resource 4. Frequencies of specific symptoms and signs in each cluster of manifestations

**Flu-like manifestations**	100.0 (37/37)
Fever	100.0 (37/37)
Headache	29.7 (11/37)
Myalgia	27.0 (10/37)
Cough	10.8 (4/37)
Rhinorrhea	5.4 (2/37)
Sore throat	13.5 (5/37)
**Gastrointestinal manifestations**	81.1 (30/37)
Anorexia	27.0 (10/37)
Nausea	64.9 (24/37)
Vomiting	29.7 (11/37)
Diarrhea	54.1 (20/37)
Constipation	2.7 (1/37)
**Hemorrhagic manifestations**	27.0 (10/37)
Head and neck hemorrhage	24.3 (9/37)
Other hemorrhage	2.7 (1/37)
**Renal manifestations**	37.8 (14/37)
Altered urine output	21.6 (8/37)
Facial or pretibial pitting edema	18.9 (7/37)
**Abdominal pain**	59.5 (22/37)
Subjective abdominal pain	43.2 (16/37)
Abdominal tenderness	35.1 (13/37)
**Back pain**	48.6 (18/37)
Subjective back pain	18.9 (7/37)
Costovertebral angle tenderness	35.1 (13/37)

Data are percentages. Data in parenthesis are numbers used to calculate percentages.
